# Rh-Catalyzed rearrangement of vinylcyclopropane to 1,3-diene units attached to *N*-heterocycles

**DOI:** 10.3762/bjoc.7.39

**Published:** 2011-03-09

**Authors:** Franca M Cordero, Carolina Vurchio, Stefano Cicchi, Armin de Meijere, Alberto Brandi

**Affiliations:** 1 Dipartimento di Chimica "Ugo Schiff", Università degli Studi di Firenze, Via della Lastruccia 13, 50019 Sesto Fiorentino (FI), Italy; 2 Institut für Organische und Biomolekulare Chemie der Georg-August-Universität Göttingen, Tammannstrasse 2, 37077 Göttingen, Germany

**Keywords:** nitrogen heterocycles, rearrangement, rhodium, small ring systems, spiro compounds

## Abstract

Dienes embedded in quinolizidine and indolizidine structures can be prepared in four steps from cyclic nitrones and bicyclopropylidene. The key intermediates α-spirocyclopropanated *N*-heterocyclic ketones, generated via a domino 1,3-dipolar cycloaddition/thermal rearrangement sequence, were converted by Wittig methylenation to the corresponding vinylcyclopropanes (VCPs), which underwent rearrangement to 1,3-dienes in the presence of the Wilkinson Rh(I) complex under microwave heating. The previously unexplored Rh(I)-catalyzed opening of the VCP moiety embedded in an azapolycyclic system occurs at high temperature (110–130 °C) to afford the corresponding 1,3-dienes in moderate yield (34–53%).

## Introduction

**Scheme 1:**
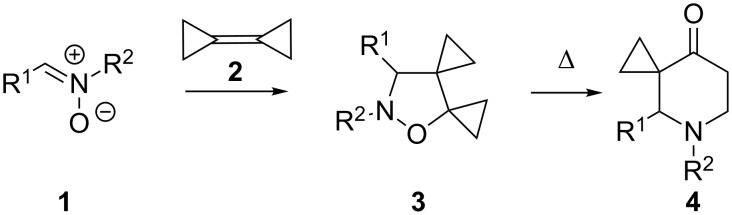
General approach to spirocyclopropanated tetrahydropyridones by 1,3-dipolar cycloaddition/thermal rearrangement.

The cyclopropyl group endows many natural and synthetic compounds with a broad spectrum of interesting properties, mainly related to its unusual bonding and inherent ring strain [1–3]. This characteristic confers on molecules containing this moiety high reactivity, especially towards ring expansion and ring-opening transformations. The smallest carbocycle can therefore be considered as a peculiar functional group that can promote unique reactivities and synthetic possibilities [4]. The main obstacle to full exploitation of this chemistry is the difficulty of selectively introducing a cyclopropyl group into a given substrate so that the various specific cyclopropane transformations can be used as a synthetic tool. In recent years we have shown that 1,3-dipolar cycloadditions of nitrones **1** to the highly strained alkene bicyclopropylidene (BCP, **2**) [5–7] afford spirocyclopropanated isoxazolidines **3** [8–9] which, on heating, rearrange [10] to yield a large variety of spirocyclopropanated heterocyclic ketones **4** depending on the nature of the starting nitrone (Scheme 1) [11–17].

This rather general and convenient access to spirocyclopropane-annelated heterocyclic ketones **4** makes it attractive for the construction of other heterocyclic compounds by selective elaboration of the α-oxocyclopropane functionality, for example, to vinylcyclopropane (VCP) by simple Wittig olefination. The rearrangement of VCPs to cyclopentenes and dienes are well known processes [18–25] that occur thermally or under catalysis by various transition metals including Rh, Ni, Pd, Cu, Cr, Mo, and Fe [26–34]. To date the metal-catalyzed rearrangement of azaheterocyclic VCP has not been reported. In the context of our interest in the VCP chemistry of spirocyclopropane-annelated heterocyclic compounds [35], we started to investigate some metal-catalyzed rearrangements. The first choice was the readily available so-called Wilkinson catalyst Rh(PPh_3_)_3_Cl, because of its documented efficiency in catalyzing the rearrangement [26] and of the possibility to extend its use to other interesting transformations, such as the [5 + 2] cycloadditions of vinylcyclopropanes to alkynes developed by Wender and co-workers [36–37]. It is known, that rhodium-catalyzed rearrangements of unactivated VCPs, without any functional substituent, usually afford dienes. In order to evaluate the influence of the *N-*heterocyclic system on the rearrangement, some model VCPs were generated by Wittig olefination of the α-oxocyclopropane group of functionalized oligocyclic spirocyclopropane-tetrahydropyridones and converted into the corresponding 1,3-dienes by treatment with Rh(PPh_3_)_3_Cl.

## Results and Discussion

**Scheme 2:**
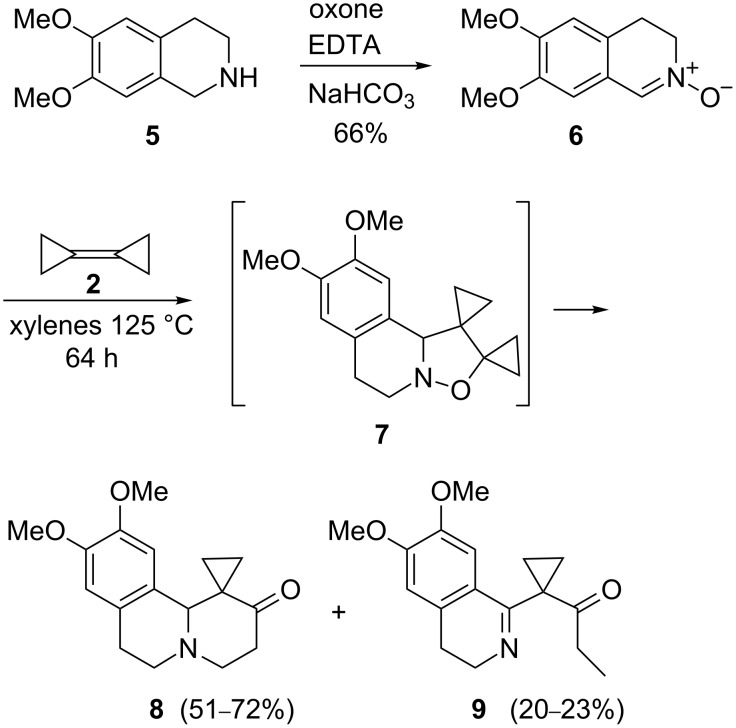
Synthesis of tetrahydrospiro[cyclopropane-1,1’(2’*H*,6’*H*)-pyrido[2,1-*a*]isoquinolin]-2’-one **8**.

The tetrahydropyridones employed in this study were prepared according to published procedures with slight modifications. In particular, oxidation of the tetrahydroquinoline **5** with oxone [38] afforded the nitrone **6** [39–41] in 66% yield (Scheme 2, see Supporting Information File 1 for full experimental data). Treatment of **6** (1.2–1.7 equiv) with BCP (**2**) in xylenes at 125 °C for 64 h directly afforded the α-oxocyclopropane derivative **8** [13] (51–72% yield) along with a minor amount of the open-chain isomer **9** (20–23% yield).

The open-chain isomer **9** is derived from a rarely observed 1,5-hydrogen shift in the cyclopropanated 1,6-diradical intermediate, which in this case is probably facilitated by the enhanced mobility of the benzylic hydrogen and by the formation of the conjugationally stabilized imine **9** [11].

**Scheme 3:**
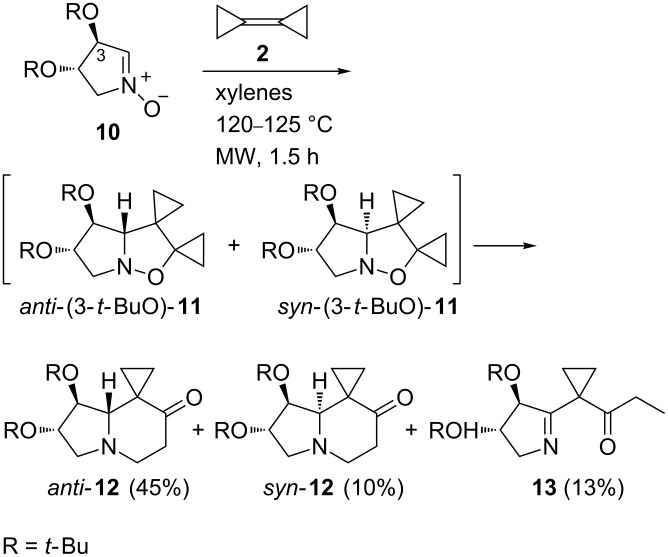
Synthesis of 7’-oxohexahydro spiro[cyclopropane-1-8’(5’*H*)indolizines] **12**.

The 1,3-dipolar cycloaddition/thermal rearrangement domino reaction of BCP (**2**) with the enantiopure nitrone **10** [42] derived from L-tartaric acid was complete within only 1.5 h at 120–125 °C under microwave (MW) heating and afforded the oxospirocyclopropanes *anti*-**12** and *syn*-**12** in 55% overall yield along with the 1,5-hydrogen shift product **13** (13%) (Scheme 3, see Supporting Information File 1 for full experimental data). The two diastereomeric indolizidinones *anti*-**12** and *syn*-**12** are formed by the thermal rearrangement of the cycloadducts *anti-*(3-*t*-BuO)-**11** and *syn-*(3-*t*-BuO)-**11**, respectively.

**Scheme 4:**
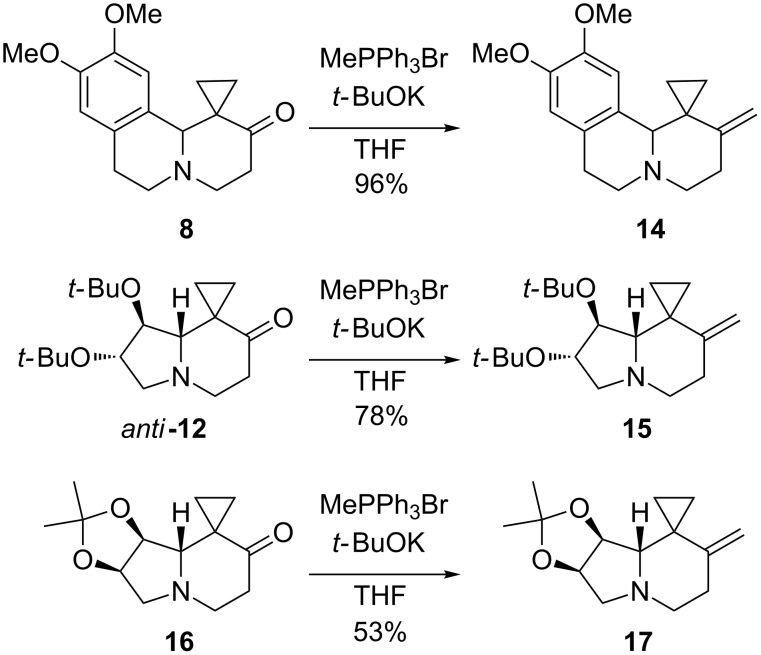
Olefination of spirocyclopropanated heterocyclic ketones **8**, **12** and **16**.

Wittig olefination of ketones **8**, *anti*-**12** and **16** [43] with MePPh_3_Br/*t*-BuOK in THF at room temperature gave the VCPs **14**, **15**, and **17** in good yields (53–96%) (Scheme 4, see Supporting Information File 1 for full experimental data). The configuration was retained under the reaction conditions in compounds **15** and **17**, as ascertained by the unique set of ^1^H NMR signals in the crude reaction mixture.

The tricyclic compound **14** was then treated with a catalytic amount of Rh(PPh_3_)_3_Cl (Table 1). In toluene at room temperature no reaction occurred, but on heating under reflux (110 °C) or at 130 °C in a microwave (MW) oven a rearrangement occurred leading to a main product assigned as the diene **18** and a mixture of the two diastereomers (*E*)- and (*Z*)-**19**. No trace of the cyclopentene-annelation product was observed in the reaction mixture. In refluxing toluene, the reaction was quite slow and after 15 h in the presence of 5 mol % of catalyst, a considerable amount of starting material still remained. Even after the addition of a second portion of the catalyst and further heating at 110 °C for 18 h, the conversion was incomplete, and the VCP **14** was recovered in 18% yield after chromatography (Table 1, entry 1). The dienes **18** and **19** were obtained in 35% overall yield (43% yield based on converted **14**). The VCP was completely consumed after 58 h at 110 °C by adding three portions of the catalyst (5 mol % each at 0, 24 and 40 h, respectively) (Table 1, entry 2). In this case, the products were obtained in a lower yield (28%) probably because they partially decomposed upon prolonged heating. The reaction carried out at 130 °C in an MW reactor for shorter times actually gave better yields (Table 1, entries 3 and 4). The best result with a conversion of 78% and 59% yield was achieved by heating a mixture of the VCP **14** and 10 mol % of the catalyst in toluene at 130 °C for 140 min (Table 1, entry 4). When the reaction was allowed to continue until all the starting material was completely consumed led to complete decomposition of the products (Table 1, entry 5). Higher temperatures (160 °C in xylenes) or the addition of AgOTf [44] did neither improve the conversion rate nor the yield of the rearrangement products (Table 1, entries 6 and 7). A slight improvement was achieved by addition of trifluoroethanol (TFE, 5% of the total volume) as a co-solvent [45]. Under these conditions, the conversion of the VCP was complete after 5.5 h at 130 °C, and the dienes **18** and **19** were obtained in a 1:1 ratio in 46% overall yield after chromatography (Table 1, entry 8, see Supporting Information File 1 for full experimental data).

**Table 1:** Rearrangement of VCP **14** catalyzed by Rh(PPh_3_)_3_Cl.

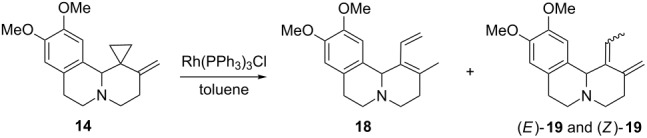						
Entry	Rh(I) (%)	Temp.^a^ (°C)	Time	Conv.^b^ (%)	**18**/**19**	Yield^c^ (%)

1	5 + 5^d^	110	15 + 18 h	82	2.5:1	35 (43)
2	5 + 5 + 5^e^	110	24 + 16 + 18 h	100	1.1:1	28 (28)
3	5	130 (MW)	1 h 30 min	62	4.5:1	46 (74)
4	10	130 (MW)	2 h 20 min	78	1.4:1	59 (75)
5	5 + 5^d^	130 (MW)	3 + 4 h	100		dec^f^
6^g^	5 + 5^d^	160 (MW)	2 + 1 h	72^h^	3.3:1	nd^i^
7^j^	5	130 (MW)	2 h	55^h^	3:1	nd^i^
8^k^	10	130 (MW)	5 h 30 min	100	1:1	46 (46)

^a^MW: the reaction was carried out in a CEM Discover microwave reactor with IR temperature monitoring. ^b^Based on recovered starting material after chromatography. ^c^Overall yield after chromatography on SiO_2_. The yield based on converted VCP is given in parentheses. ^d^The catalyst was added in two batches (5 mol % each at 0 and 15 h in entry 1, at 0 and 3 h in entry 5, at 0 and 2 h in entry 6). ^e^The catalyst was added in three batches (5 mol % each at 0, 24 and 40 h). ^f^Decomposition products. ^g^The reaction was run in xylenes. ^h^Determined by ^1^H NMR analysis of the crude reaction mixture. ^i^Not determined. ^j^5% AgOTf was added. ^k^5% TFE was added.

The collected data show that longer reaction times significantly influence the product ratio in favour of the dienes **19** (Table 1). These results are in accord with an isomerization of **18** into **19** under the reaction conditions. However, in the absence of the catalyst, heating of diene **18** under otherwise identical conditions did not induce any isomerization of **18** to **19**, which confirms that Rh also catalyzes the 1,5-hydrogen shift in **18**.

**Figure 1:**
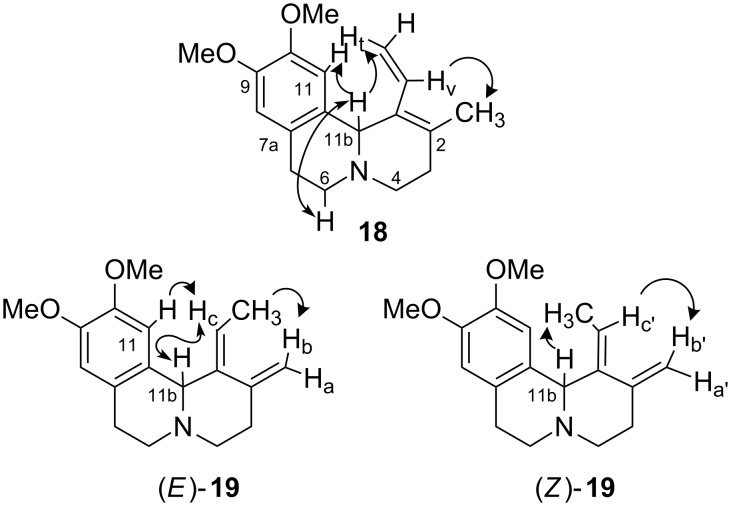
Key NOE interactions. **18**: 11b-H/11-H, 11b-H/6-H, 11b-H/H_t_, H_v_/2-CH_3_; *E*-**19**: H_b_/CH_3_, H_c_/11b-H, H_c_/11-H; *Z*-**19**: H_b’_/H_c’_, 11b-H/CH_3_.

The structure assignment was easily made on the basis of ^1^H NMR data. In particular, the diene **18** showed the typical signals of an exocyclic vinyl substituent (δ 7.00 (=C*H_v_*), 5.17 (=CH*H_cis_*), 5.17 (=CH*H_trans_*) ppm; *J_trans_* = 17.5, *J_cis_* = 11.3 Hz). Irradiation of 11b-H (δ 4.76 ppm) produced a positive NOE on the olefinic *H_trans_* whereas irradiation of =CH resulted in enhancement of the 2-C*H*_3_ signal (δ 1.87 ppm) suggesting a preferred *s-trans* conformation for the diene moiety in solution (Figure 1).

The ^1^H NMR spectra of the dienes **19** showed a quartet in the region of olefinic due to the resonance of the proton H_c_ coupled with the methyl group (δ 5.29 and 5.96 ppm, ^3^*J* = 6.9–7.0 Hz) and two signals due to the methylene protons H_a_ and H_b_ (δ 5.06 and 4.75; 4.89 and 4.65 ppm, *^2^J* = 2.3–2.1 Hz).

The *E*- and *Z*-configuration of the dienes **19** was determined by NOESY 1D NMR spectroscopy. In the major compound (*E)*-**19**, irradiation of the olefinic H_b_ (δ 4.75 ppm) gave enhancement of the ethylidene methyl group (δ 1.76 ppm), and irradiation of H_c_ (δ 5.29 ppm) showed enhancement at 11-H and 11b-H (δ 6.56 and 4.41 ppm). For the isomer *(Z)*-**19**, irradiation of H_c’_ (δ 5.96 ppm) gave enhancement of H_b’_ (δ 4.89 ppm) and an NOE interaction was present between the methyl group and 11b-H (δ 5.02 ppm) in agreement with the assigned configuration (Figure 1).

**Scheme 5:**
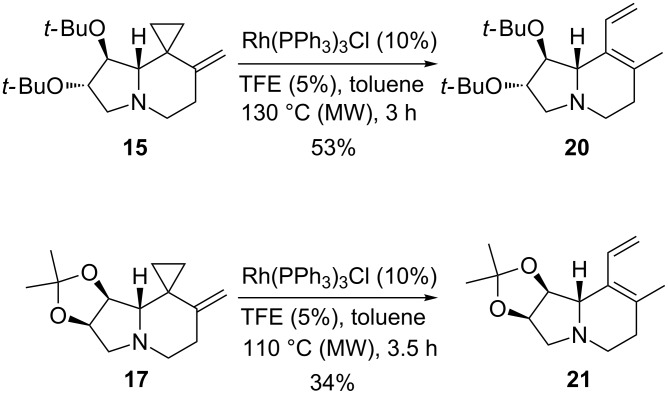
Rearrangement of VCPs **15** and **17** catalyzed by Rh(PPh_3_)_3_Cl.

The VCPs **15** and **17** were completely consumed on heating in toluene in a MW oven at 130 °C for 3 h and 110 °C for 3.5 h, respectively, in the presence of Rh(PPh_3_)_3_Cl (10%) and TFE (5%). In these cases, dienes **20** and **21** were obtained in 53 and 34% yield, respectively, as the sole reaction products (Scheme 5, see Supporting Information File 1 for full experimental data). Their structures were assigned analogously as before. Compounds **20** and **21** were found to be unstable upon standing for prolonged periods, even at low temperatures. This explains the low isolated yields in their syntheses.

**Scheme 6:**
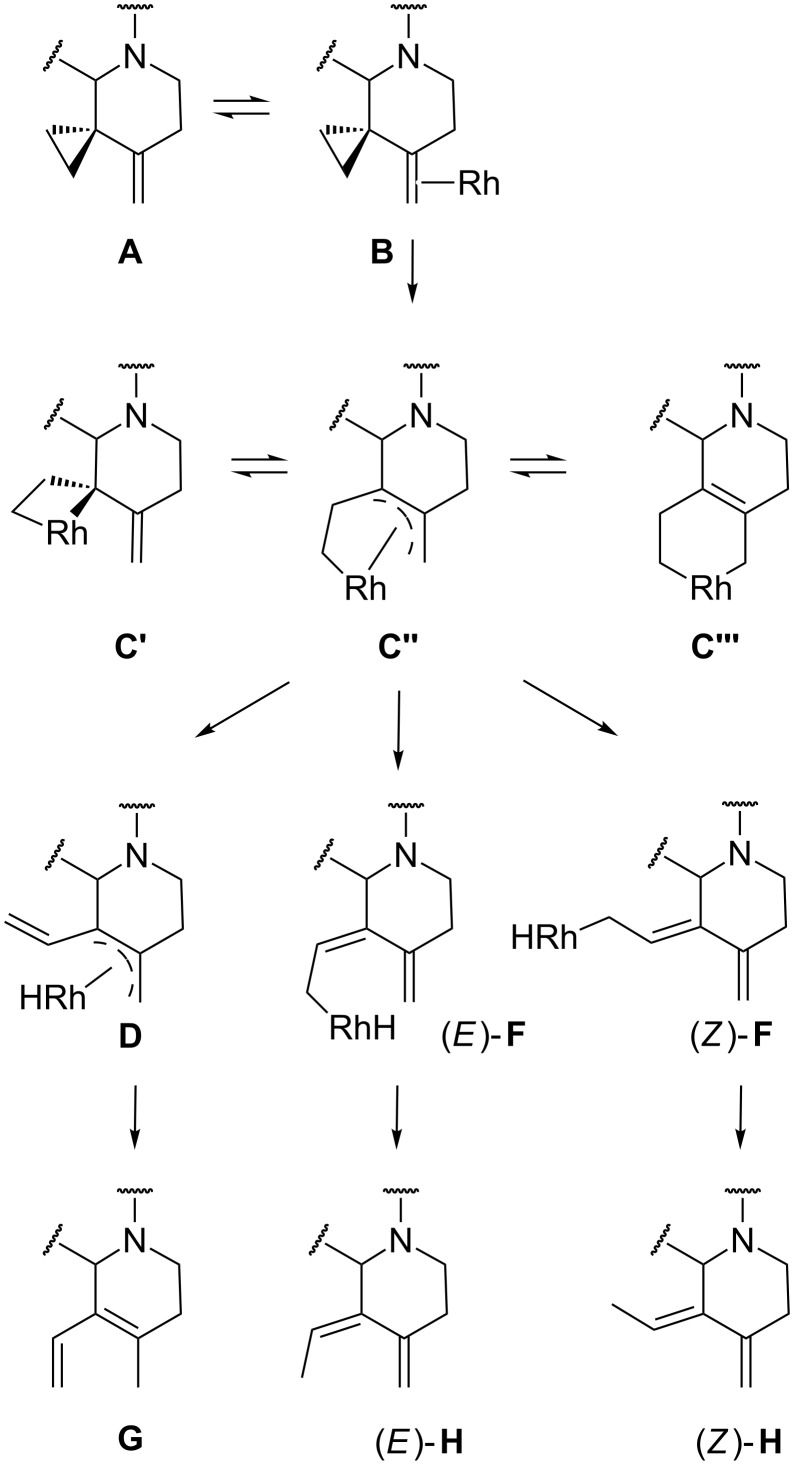
Mechanism of the rearrangement of heterocyclic VCPs catalyzed by Rh(PPh_3_)_3_Cl.

Analogously to other Rh(I)-catalyzed VCP rearrangements [46–47], the mechanism of the rearrangement likely involves insertion of the Rh(I) species into the cyclopropane ring of the VCP system, with or without incorporation of the double bond to form the intermediates **C** which can undergo metal hydride elimination or 1,3-hydride migration to the rhodium to give, respectively, the allyl- and alkylrhodium(III) hydride complexes **D** and **F**. Metal extrusion by reductive elimination leads to the observed dienes and regeneration of the catalyst (Scheme 6).

## Conclusion

Azaheterocycles **14**, **15** and **17** containing a spiro-annelated VCP moiety have been synthesized starting from cyclic nitrones and BCP by a three-step two-pot sequence consisting of a 1,3-dipolar cycloaddition, thermal rearrangement and Wittig methylenation. These compounds in the presence of the Wilkinson Rh(I) complex at high temperatures (110–130 °C) under MW heating underwent a slow rearrangement to afford the corresponding azaheterocycles containing 1,3-diene units in moderate yields. The rearrangement produced mixtures of isomeric dienes from benzoquinolizidine **14** and was regioselective in the case of indolizidines **15** and **17**, showing that the Wilkinson Rh(I) catalyst is also capable of inducing the VCP rearrangement in the presence of strongly nucleophilic azaheterocycles. Accordingly, new functionalised heterocyclic compounds can be produced by the straightforward methodology based on nitrones and bicyclopropylidene.

## Supporting Information

Supporting information features experimental procedures and spectroscopic data.

File 1 Experimental part.
