# Supplementary material for: Rh-Catalyzed rearrangement of vinylcyclopropane to 1,3-diene units attached to N-heterocycles
Source: Beilstein J Org Chem. 2011 Mar 9;7:298–303. doi: 10.3762/bjoc.7.39 (PMC3063054; doi:10.3762/bjoc.7.39)
Supplement: File 1 — Experimental part. [file Beilstein_J_Org_Chem-07-298-s001.pdf]

Supporting Information

for

Rh-Catalyzed rearrangement of vinylcyclopropane to 1,3-diene units attached to *N*-heterocycles

Franca M. Cordero*[1], Carolina Vurchio[1], Stefano Cicchi[1], Armin de Meijere[2] and Alberto Brandi*[1]

Address: [1]Dipartimento di Chimica "Ugo Schiff", Università degli Studi di Firenze, Via della Lastruccia 13, 50019 Sesto Fiorentino (FI), Italy and [2]Institut für Organische und Biomolekulare Chemie der Georg-August-Universität Göttingen, Tammannstrasse 2, 37077 Göttingen, Germany

Email: Franca M. Cordero* - franca.cordero@unifi.it; Carolina Vurchio - carla.vurchio@unifi.it; Armin de Meijere - ameijer1@gwdg.de; Alberto Brandi* - alberto.brandi@unifi.it

* Corresponding author

Experimental part

Table of contents

General remarks S2

6,7-Dimethoxy-3,4-dihydroisoquinoline 2-oxide (**6**) S2

9’,10’-Dimethoxy-3’,4’,7’,11b’-tetrahydro-spiro[cyclopropane-1,1’(2’*H*,6’*H*)-pyrido[2,1-*a*]isoquinolin]-2’-one (**8**) and 1-[1-(6,7-dimethoxy-3,4-dihydro-1-isoquinolinyl)cyclopropyl]-1-propanone (**9**) S3

(1’*S*,2’*S*,8a’*S*)-1’,2’-Di-tert-butoxy-7’-oxohexahydrospiro[cyclopropane-1-8’(5’*H*)indolizine] (*anti*-**12**), (1’*S*,2’*S*,8a’*R*)-1’,2’-di-*tert*-butoxy-7’-oxohexahydrospiro [cyclopropane-1-8’(5’*H*)indolizine] (*syn*-**12**), and 1-{1-[(3*S*,4*S*)-3,4-di-*tert*-butoxy-3,4-dihydro-2H-pyrrol-5-yl]cyclopropyl}-1-propanone (**13**) S4

9’,10’-Dimethoxy-2’-methylene-3’,4’,7’,11b’-tetrahydro-spiro[cyclopropane-1,1’(2’*H*,6*H*’)-pyrido[2,1-*a*]isoquinoline] (**14**) S5

(1'*S*,*2'S*,8a'*S*)-1',2'-Di-*tert*-butoxy-7'-methylenehexahydrospiro[cyclopropane-1-8'(5'*H*)indolizine] (**15**) S6

(3a'*R*,9a'*S*,9b'*S*)-2',2'-Dimethyl-8'-methylenehexahydrospiro{cyclopropane-1-9'(6'*H*)[1',3']dioxolo[4',5'-*a*]indolizine} (**17**) S6

9,10-Dimethoxy-2-methyl-1-vinyl-3,6,7,11b-tetrahydro-4*H*-pyrido[2,1-*a*]isoquinoline (**18**), (1*E*)-1-ethylidene-9,10-dimethoxy-2-methylene-1,3,4,6,7,11b-hexahydro-2*H*-pyrido[2,1-*a*]isoquinoline [(*E*)-**19**] and (1*Z*)-1-ethylidene-9,10-dimethoxy-2-methylene-1,3,4,6,7,11b-hexahydro-2*H*-pyrido[2,1-*a*]isoquinoline [(*Z*)-**19**] S7

(1*S*,2*S*,8a*S*)-1,2-Di-*tert*-butoxy-7-methyl-8-vinyl-1,2,3,5,6,8a-hexahydroindolizine (**20**) S8

(3a*R*,9a*S*,9b*S*)-2,2,8-Trimethyl-9-vinyl-3a,4,6,7,9a,9b-hexahydro[1,3]dioxolo[4,5-*a*]indolizine (**21**) S8

General Remarks: All reactions requiring anhydrous conditions were carried out under a nitrogen, atmosphere and the solvents were dried appropriately before use. R_f values refer to TLC on 0.25 mm silica gel plates. Microwave-assisted reactions were carried out in a CEM Discover (TM) single mode microwave reactor with an IR temperature sensor. $CDCl_3$ was used as solvent for NMR measurements. NMR data are reported in δ (ppm) from TMS at 25 °C and peak assignments were made on the basis of ^1H–^1H COSY and HMQC experiments. IR spectra were recorded in $CDCl_3$ solution unless otherwise specified. Accurate mass spectra were recorded on a LTQ-Orbitrap high-resolution mass spectrometer (Thermo, San Jose, CA, USA), equipped with a conventional ESI source. Bicyclopropylidene (BCP) was prepared according to the previously published procedure.[1]

*6,7-Dimethoxy-3,4-dihydroisoquinoline 2-oxide (**6**)*

$NaHCO_3$ (5.55 g, 66 mmol) was added to a stirred suspension of isoquinoline **5** (2.55 g, 13.2 mmol) in a 4:1 mixture of MeCN/THF (24 mL) and aqueous Na_2EDTA (0.01 M, 18.4 mL). The mixture was then cooled in an ice bath and Oxone® (10 g, 16.25 mmol) added portionwise over 4.5 h. The mixture was stirred at 0 ºC for 45 min, then diluted with EtOAc (20 mL) and H_2O (20 mL). The two phases were separated and the aqueous solution extracted with CH_2Cl_2 (4 x 20 mL). The combined organic phases were dried over Na_2SO_4, filtered and concentrated under reduced pressure to afford the crude nitrone. Recrystallization from CH_2Cl_2 /petroleum ether and chromatography on silica gel (eluent: EtOAc/MeOH, first 2:1, then 3:2) of the compound recovered from the recrystallization mother liquors gave analytically pure **6** (1.83 g, 66%) as a yellow solid identical to that reported in the literature.[2]

6: R_f 0.2 (EtOAc/MeOH 2:1); m.p. 182–185 °C; ^{1}H NMR (400 MHz): δ = 7.66 (s, 1H, 1-H), 6.72 (s, 1H) and 6.61 (s, 1H) (5-H and 8-H), 4.07 (pseudo t, 2H, *J* = 7.9 Hz, 3-H), 3.90 (s, 3H, OC*H*$_3$), 3.88 (s, 3H, OC*H*$_3$), 3.11 (t, 2H, *J* = 7.9 Hz, 4-H) ppm. ^{13}C NMR (100 MHz): δ = 149.9 (s) and 148.4 (s) (C-6 and C-7), 133.7 (d, C-1), 123.2 (s) and 121.0 (s) (C-4a and C-7a), 110.7 (d) and 108.5 (d) (C-5 and C-8), 57.7 (t, C-3), 56.2 (q, O*C*H$_3$), 56.1 (q, O*C*H$_3$), 27.6 (t, C-4) ppm. IR (KBr): ν = 3032, 2923, 1598, 1598, 1517, 1282, 1227, 1164, 1119 cm^{-1}. MS (70 eV, EI): *m*/z (%) = 207 (100) [M$^+$], 192 (35), 176 (9), 163 (8), 146 (13), 133 (35). $C_{11}H_{13}NO_3$ (207.23): calcd. C 63.76, H 6.32, N 6.76; found C 63.41, H 6.51, N 6.69.

*9’,10’-Dimethoxy-3’,4’,7’,11b’-tetrahydro-spiro[cyclopropane-1,1’(2’H,6’H)-pyrido[2,1-a]isoquinolin]-2’-one (**8**) and 1-[1-(6,7-dimethoxy-3,4-dihydro-1-isoquinolinyl)cyclopropyl]-1-propanone (**9**)*

A mixture of the nitrone **6** (72 mg, 0.35 mmol) and BCP **2** (0.03 mL, 0.29 mmol) in xylenes (2.2 mL) was heated in a screw-cap sealed Sovirel tube at 125 °C for 64 h. The reaction mixture was filtered through a short pad of silica gel eluting first with petroleum ether to remove the solvent and then with MeOH. Chromatography on silica gel (eluent: EtOAc/MeOH 3:1) of the crude mixture afforded **8**[3] (60 mg, 72%) as a pale yellow oil [R_f 0.35 (EtOAc/MeOH 3:1)] and **9** as a beige solid (19 mg, 23%). The same procedure repeated on a larger scale (**6**: 750 mg, 3.62 mmol; **2**: 0.2 mL, 2.18 mmol; xylenes: 16.4 mL) afforded **8** and **9** in lower yield (**8**: 322 mg, 51%; **9**: 126 mg, 20%).

9: R_f 0.63 (EtOAc/MeOH 3:1); m.p. 75–77 °C, ^{1}H NMR (400 MHz): δ = 6.99 (s, 1H, 8-H), 6.71 (s, 1H, 5-H), 3.91 (s, 3H, OC*H*$_3$), 3.80 (s, 3H, OC*H*$_3$), 3.73–3.67 (m, 2H, 3-H), 2.68 (pseudo t, *J* = 7.2 Hz, 2H, 4-H), 2.35 (q, *J* = 7.2 Hz, 2H, C*H*$_2$Me), 1.59–1.51 (m, 2H, *c*Pr), 1.36–1.28 (m, 2H, *c*Pr), 0.89 (t, *J* = 7.2 Hz, 3H, CH$_2$C*H*$_3$) ppm. ^{13}C NMR (100 MHz): δ = 209.5 (s, *C*O), 164.6 (s, C-1), 151.1 (s, C-7), 147.7 (s, C-6), 131.4 (s, C-8a), 121.9 (s, C-4a), 110.4 (d, C-5), 108.7 (d, C-8), 56.1 (q, O*C*H$_3$), 56.0 (q, O*C*H$_3$), 47.4 (t, C-3), 37.7 (s, *c*Pr), 34.0 (t, *C*H$_2$Me), 25.3 (t, C-4), 16.7 (t, 2C, *c*Pr), 8.0 (q, CH$_2$*C*H$_3$) ppm. IR: ν = 3005, 2933, 1689, 1622, 1517, 1362, 1274, 1136 cm^{-1}. MS (70 eV, EI): *m*/z (%) = 287 (86) [M$^+$], 286 (62), 272 (96), 256 (30), 242 (19), 230 (58), 216 (65), 200 (100). $C_{17}H_{21}NO_3$ (287.35): calcd. C 71.06, H 7.37, N 4.87; found C 70.66, H 7.73, N 4.89.

*(1’S,2’S,8a’S)-1’,2’-Di-tert-butoxy-7’-oxohexahydrospiro[cyclopropane-1-8’(5’H)indolizine] (anti-**12**), (1’S,2’S,8a’R)-1’,2’-di-tert-butoxy-7’-oxohexahydrospiro[cyclopropane-1-8’(5’H)indolizine] (syn-**12**), and 1-{1-[(3S,4S)-3,4-di-tert-butoxy-3,4-dihydro-2H-pyrrol-5-yl]cyclopropyl}-1-propanone (**13**)*

A mixture of the nitrone **10** (507 mg, 2.21 mmol) and BCP **2** (0.23 mL, 2.43 mmol) in xylenes (4 mL) in a sealed vial was irradiated in a microwave reactor first at 120 °C for 1 h and then at 125 °C for 30 min. The reaction mixture was filtered through a short pad of silica gel eluting first with petroleum ether to remove the solvent and then with MeOH. Chromatography on silica gel (eluent: EtOAc/MeOH from 20:1 to 5:1) of the concentrated methanolic solution afforded *anti*-**12** (308 mg, 45%) as a pale yellow oil, *syn*-**12** (69 mg, 10%) and **13** (88 mg, 13%). Compound *anti*-**12** had the same spectral characteristics as its enantiomer,[3] but had the opposite sign of optical rotation.

anti-**12**: R_f 0.50 (EtOAc/MeOH 10:1); $[\alpha]_D^{22}$= +17.6 (*c* = 0.68, $CHCl_3$); ^{1}H NMR (400 MHz): δ = 3.91 (dt, *J* = 6.1, 2.5 Hz, 1H, 2-H), 3.62 (dd, *J* = 6.4, 2.5 Hz, 1H, 1-H), 3.21–3.15 (m, 1H, 5-H_a), 3.07 (dd, *J* = 10.3, 2.5 Hz, 1H, 3-H_a), 2.82–2.65 (m, 4H, 3-H_b + 5-H_b + 6-H_a + 8a-H), 2.46–2.33 (m, 1H, 6-H_b), 1.43–1.35 (m, 1H, *c*-Pr), 1.34–1.28 (m, 1H, *c*-Pr), 1.21 (s, 9H, *t*Bu), 1.20 (s, 9H, *t*Bu), 0.99–0.86 (m, 2H, *c*-Pr) ppm. ^{13}C NMR (50 MHz): δ = 208.4 (s, CO), 81.9 (d, C-1), 78.0 (d, C-2), 74.6 (s, *C*Me_3), 74.1 (s, *C*Me_3), 68.8 (d, C-8a), 60.2 (t, C-3), 48.8 (t, C-5), 37.2 (t, C-6), 30.1 (s, C-8), 29.0 (q, 3C, CH_3), 28.9 (q, 3C, CH_3), 15.4 (t, *c*Pr), 13.6 (t, *c*Pr) ppm.

syn-**12**: R_f 0.17 (EtOAc/MeOH 10:1); $[\alpha]_D^{23}$= –170.6 (*c* = 0.835, $CHCl_3$); ^{1}H NMR (400 MHz): δ = 3.80 (d, *J* = 3.0 Hz, 1H, 2-H), 3.51 (d, *J* = 3.0 Hz, 1H, 1-H), 3.41 (dt, *J* = 2.3; 13.5 Hz, 1H, 5-H_a), 3.13 (dd, *J* = 9.9, 3.6 Hz, 1H, 3-H_a), 3.07 (d, *J* = 3.5 Hz, 1H, 8a-H), 2.94 (d, *J* = 10.0 Hz, 1H, 3-H_b), 2.89 (ddd, *J* = 14.1, 4.8, 2.4 Hz, 1H, 5-H_b), 2.75 (ddd, *J* = 17.2, 12.9, 4.8 Hz, 1H, 6-H_a), 2.10 (dt, *J* = 17.2, 2.3 Hz, 1H, 6-H_b), 1.66 (ddd, *J* = 9.5, 6.7, 4.3 Hz, 1H, *c*-Pr), 1.19 (s, 9H, *t*Bu), 1.12 (s, 9H, *t*Bu), 1.01–0.94 (m, 1H, *c*-Pr), 0.87 (ddd, *J* = 9.5, 6.5, 3.3 Hz, 1H, *c*-Pr), 0.59 (ddd, *J* = 9.0, 6.5, 4.4 Hz, 1H, *c*-Pr) ppm. ^{13}C NMR (50 MHz): δ = 209.9 (s, CO), 77.3 (d, C-1), 75.6 (d, C-2), 74.4 (s, *C*Me_3), 73.7 (s, *C*Me_3), 68.4 (d, C-8a), 58.2 (t, C-3), 45.6 (t, C-5), 35.9 (t, C-6), 28.5 (q, 3C, CH_3), 28.2 (q, 3C, CH_3), 26.4 (s, C-8), 21.4 (t, *c*Pr), 9.2 (t, *c*Pr) ppm. IR: ν = 3068, 2959, 1697, 1368, 1187, 1078 cm^{-1}. MS (70 eV, EI): *m*/z (%) = 309 (3) [M^+], 252 (95), 236 (4), 196 (40), 137 (44), 57 (100).

13: R_f 0.85 (EtOAc/MeOH 10:1); $[\alpha]_D^{20}$= –4.55 (*c* = 0.84, $CHCl_3$); ^{1}H NMR (400 MHz): δ = 4.50–4.46 (m, 1H, 4-H), 4.14–4.03 (m, 2H, 3-H + 2-H_a), 3.49 (ddd, *J* = 14.9, 4.9, 1.2 Hz, 1H, 2-H_b), 2.74 (dq, *J* = 18.0; 7.2 Hz, 1H, CH*H*Me), 2.53 (dq, *J* = 18.0; 7.2 Hz, 1H, CH*H*Me), 1.58 (ddd, *J* = 9.4, 7.1, 3.8 Hz, 1H, *c*-Pr), 1.38 (ddd, *J* = 9.3, 7.1, 3.7 Hz, 1H, *c*-Pr),1.18 (s, 9H, *t*Bu), 1.16 (s,

9H, *t*Bu), 1.10 (ddd, *J* = 9.4, 7.0, 3.7 Hz, 1H, *c*-Pr), 1.00 (t, *J* = 7.2 Hz, 3H, CH_2CH_3), 0.87 (ddd, *J* = 9.3, 7.0, 3.8 Hz, 1H, *c*-Pr) ppm. ^{13}C NMR (50 MHz): δ = 207.4 (s, CO), 176.4 (s, C=N), 84.0 (d, C-4), 79.5 (d, C-3), 75.2 (s, CMe_3), 73.8 (s, CMe_3), 65.3 (t, C-2), 34.4 (t, CH_2Me), 33.7 (s, *c*Pr), 28.7 (q, 9C, *t*-Bu), 18.0 (t, *c*Pr), 13.5 (t, *c*Pr), 8.1 (q, CH_2CH_3) ppm. IR: ν = 2968, 1697, 1634, 1368, 1187, 1069 cm^{-1}. MS (70 eV, EI): *m*/z (%) = 309 (1) [M$^+$], 253 (3), 197 (13), 168 (10), 124 (5), 57 (100). $C_{18}H_{31}NO_3$ (309.44): calcd. C 69.86, H 10.10, N 4.53; found C 69.53, H 10.21, N 4.63.

*9',10'-Dimethoxy-2'-methylene-3',4',7',11b'-tetrahydro-spiro[cyclopropane-1,1'(2'H,6H')-pyrido[2,1-a]isoquinoline] (**14**)*

A suspension of methyl(triphenyl)phosphonium bromide (1.29 g, 3.6 mmol) in THF (18.4 mL) was treated with *t*-BuOK (387 mg, 3.45 mmol). A solution of the ketone **8** (416 mg, 1.44 mmol) in THF (16.6 mL) was then added dropwise over 20 min to the yellow suspension. The reaction mixture was stirred for 18 h at r.t., diluted with H_2O (30 mL) and the THF evaporated under reduced pressure. After extraction with Et_2O (3 x 20 mL) , the combined organic layers were dried over Na_2SO_4, filtered and evaporated. The residue was purified by chromatography on silica gel (eluent: Et_2O/MeOH 4:1) to afford the VCP **14** (395 mg, 96%) as a pale yellow solid.

14: R_f 0.26 (Et_2O/MeOH 3:1); m.p. 91–93 °C; ^{1}H NMR (400 MHz): δ = 6.95 (s, 1H) and 6.54 (s, 1H) (8'-H and 11'-H), 4.67 (br s, 1H, =CH*H*), 4.66 (br s, 1H, =CH*H*), 3.83 (s, 3H, OCH_3), 3.82 (s, 3H, OCH_3), 3.49 (s, 1H, 11b'-H), 3.40 (ddd, *J* = 13.1, 6.6, 3.7 Hz, 1H, 6'-H_a), 3.13 (ddd, *J* = 13.1, 9.4, 6.6 Hz, 1H, 6'-H_b), 2.96–2.83 (m, 3H, 4'-H + 7'-H_a), 2.58 (ddd, *J* = 16.8, 6.4, 3.7 Hz, 1H, 7'-H_b), 2.48–2.38 (m, 1H, 3'-H_a), 2.33 (dt, *J* = 13.0, 4.5 Hz, 1H, 3'-H_b), 1.14–1.04 (m, 1H, *c*Pr), 0.75–0.66 (m, 2H, *c*Pr), 0.57–0.47 (m, 1H, *c*Pr) ppm. ^{13}C NMR (100 MHz): δ = 147.6 (s), 147.4 (s) and 146.5 (s) (C-2', C-9', and C-10'), 126.8 (s) and 126.7 (s) (C-7a' and C-11a'), 111.4 (d) and 111.1 (d, C-8' and C-11'), 106.9 (t, =CH_2) 2, 65.1 (d, C-11b'), 55.8 (q, OCH_3), 55.7 (q, OCH_3), 48.9 (t, 2C, C-4' + C-6'), 33.0 (t, C-3'), 26.0 (s, C-1), 24.3 (t, C-7'), 10.8 (t, 2C, C-2 + C-3) ppm. IR: ν = 3081, 3000, 2936, 2846, 1605, 1514, 1460, 1261 cm^{-1}. MS (70 eV, EI): *m*/z (%) = 285 (78) [M$^+$], 284 (100), 270 (38), 256 (12), 242 (12), 218 (27), 203 (46), 190 (86). $C_{18}H_{23}NO_2$ (285.38): calcd. C 75.76, H 8.12, N 4.91; found C 75.67, H 8.01, N 4.89

(1'S,2'S,8a'S)-1',2'-Di-tert-butoxy-7'-methylenehexahydrospiro[cyclopropane-1-8'(5'H)indolizine] ***(15)***

The VCP **15** was prepared starting from the ketone *anti*-**12** (280 mg, 0.9 mmol) following the same procedure used to prepare **14** (3 h instead of 18 h). Chromatography on silica gel (eluent: EtOAc/MeOH, first 14:1, then 10:1) afforded **15** (215 mg, 78%) as a yellow oil.

15: R_f 0.26 (EtOAc/MeOH 15:1); $[\alpha]_D^{27}$= +28.7 (*c* = 0.985, CH_2Cl_2); ^{1}H NMR (200 MHz): δ = 4.68–4.65 (m, 1H, =C*H*H), 4.61 (d, *J* = 1.8 Hz, 1H, =C*H*H), 3.89 (dt, *J* = 6.5, 2.8 Hz, 1H, 2-H), 3.64 (dd, *J* = 7.2, 2.9 Hz, 1H, 1-H), 3.11–2.98 (m, 1H, 5-H_a), 2.99 (dd, *J* = 10.8, 2.6 Hz, 1H, 3-H_a), 2.75 (dd, *J* = 10.8, 6.5 Hz, 1H, 3-H_b), 2.59–2.41 (m, 2H, 5H_b + 6H_a), 2.34 (d, *J* = 7.2 Hz, 1H, 8a-H), 2.31–2.20 (m, 1H, 6-H_b), 1.22 (s, 9H, C*H*$_3$), 1.18 (s, 9H, C*H*$_3$), 1.06–0.95 (m, 1H, *c*Pr), 0.82–0.61 (m, 2H, *c*Pr), 0.52–0.42 (m, 1H, *c*Pr) ppm. ^{13}C NMR (50 MHz): δ = 148.0 (s, C-7), 106.4 (t, =*C*H_2), 80.4 (d, C-1), 79.2 (d, C-2), 74.4 (s, *C*Me_3), 73.8 (s, *C*Me_3), 70.4 (d, C-8), 60.1 (t, C-3), 52.2 (t, C-5), 32.8 (t, C-6), 29.2 (q, 3C, CH_3), 29.1 (q, 3C, CH_3), 23.5 (s, C-8), 9.7 (t, *c*Pr), 9.4 (t, *c*Pr) ppm. IR: ν = 3077, 2968, 1648, 1368, 1191, 1074 cm^{-1}; MS (70 eV, EI): *m*/z (%) = 306 (2) $[M–1]^+$, 250 (100), 234 (7), 194 (31), 135 (33), 57 (67). HRMS: calcd for $C_{19}H_{34}NO_2$ $[M+H]^+$ 308.25841, found 308.25819.

(3a'R,9a'S,9b'S)-2',2'-Dimethyl-8'-methylenehexahydrospiro{cyclopropane-1-9'(6'H)[1',3']dioxolo[4',5'-a]indolizine} ***(17)***

The VCP **17** was prepared starting from the ketone **16** (222 mg, 0.936 mmol) following the same procedure used to prepare **14**. Chromatography on silica gel (eluent: CH_2Cl_2/MeOH 15:1) afforded **17** (118 mg, 53%) as a yellow oil.

17: R_f 0.33 (CH_2Cl_2/MeOH 15:1); $[\alpha]_D^{27}$= +26.6 (*c* = 0.662, CH_2Cl_2); ^{1}H NMR (200 MHz): δ = 4.74–4.61 (m, 3H), 3.90 (pseudo t, *J* = 7.0 Hz), 3.43 (dd, *J* = 9.6, 6.4 Hz, 1H), 3.15–3.06 (m, 1H), 2.48 (d, *J* = 6.4 Hz, 1H), 2.45–2.21 (m, 4H), 1.48 (s, 3H), 1.28 (s, 3H), 1.07–0.81 (m, 1H), 0.74-0.62 (m, 1H), 0.31–0.18 (m, 1H) ppm. ^{13}C NMR (50 MHz): δ = 148.0 (s, C-8), 114.0 (s, C-2), 106.2 (t, =*C*H_2), 79.3 (d, C-9b), 77.6 (d, C-3a), 72.0 (d, C-9a), 60.1 (t, C-4), 53.3 (t, C-6), 33.8 (t, C-7), 27.2 (q, CH_3), 25.2 (q, CH_3), 24.8 (s, C-9), 9.9 (t, *c*Pr), 6.2 (t, *c*Pr) ppm. IR: ν = 3081, 2933, 1650, 1383, 1375, 1158, 1120 cm^{-1}; MS (70 eV, EI): *m*/z (%) = 235 (21) $[M^+]$, 234 (100), 220 (8), 207 (4), 176 (77), 135 (74), 120 (84), 107 (44), 93 (57), 79 (65). HRMS: calcd for $C_{14}H_{22}NO_2$ $[M+H]^+$ 236.16451, found 236.16437.

*9,10-Dimethoxy-2-methyl-1-vinyl-3,6,7,11b-tetrahydro-4H-pyrido[2,1-a]isoquinoline (**18**), (1E)-1-ethylidene-9,10-dimethoxy-2-methylene-1,3,4,6,7,11b-hexahydro-2H-pyrido[2,1-a]isoquinoline [(E)-**19**] and (1Z)-1-ethylidene-9,10-dimethoxy-2-methylene-1,3,4,6,7,11b-hexahydro-2H-pyrido[2,1-a]isoquinoline [(Z)-**19**]*

(Table 1, entry 8): A mixture of VCP **14** (33 mg, 0.116 mmol), $Rh(PPh_3)_3Cl$ (10.7 mg, 0.012 mmol) and TFE (0.1 mL; degassed with N_2 prior to use) in toluene (1.9 mL; distilled from Na/benzophenone and degassed with N_2 prior to use) in a sealed vial was irradiated in a microwave reactor at 130 °C for 5 h and 30 min. Chromatography on silica gel (eluent: Et_2O/MeOH 6:1) of the concentrated crude mixture afforded **18** (8.0 mg, 24%) and a mixture of (*E*)-**19** and (*Z*)-**19** (7.2 mg, 22%).

18: R_f 0.28 (Et_2O/MeOH 3:1); ^{1}H NMR (400 MHz): δ = 7.00 (dd, *J* = 17.5, 11.3 Hz, 1H, =C*H*), 6.88 (s, 1H, 11-H), 6.56 (s, 1H, 8-H), 5.17 (d, *J* = 11.3 Hz, 1H, =CH*H*), 5.15 (d, *J* = 17.5 Hz, 1H, =CH*H*), 4.76 (br s, 1H, 11b-H), 3.84 (s, 3H, OCH_3), 3.75(s, 3H, OCH_3), 3.55 (ddd, *J* = 13.6, 11.3, 6.4 Hz, 1H, 6-H_a), 3.17 (ddd, *J* = 13.6, 7.0, 2.2 Hz, 1H, 6-H_b), 2.96 (dddm, *J* = 16.8, 11.3, 7.0 Hz, 1H, 7-H_a), 2.81 (dt, *J* = 5.1, 11.2 Hz, 1H, 4-H_a), 2.66 (dd, *J* = 11.3, 7.5 Hz, 1H, 4-H_b), 2.62–2.45 (m, 2H, 7-H_b + 3-H_a), 2.04 (br dd, *J* = 18.5, 5.0 Hz, 1H, 3-H_b), 1.87 (br s, 3H, 2-CH_3) ppm. ^{13}C NMR (50 MHz): δ = 147.2 (s) and 146.7(s) (C-9 and C-10), 135.0 (d, =*C*H), 133.8 (s), 130.7 (s), 129.0 (s), and 125.8 (s) (C-1, C-2, C-7a, and C-11a), 112.5 (t, =CH_2), 112.4 (d, C-11), 111.2 (d, C-8), 55.8 (q, 2C, O*C*H_3 x 2), 55.7 (d, C-11b), 50.9 (t, C-6), 42.6 (t, C-4), 33.2 (t, C-3), 23.3 (t, C-7), 18.9 (q, 2-*C*H_3) ppm. IR: ν = 3089, 3008, 2936, 2854, 1605, 1514, 1465, 1258, 1216 cm^{-1}. MS (70 eV, EI): *m*/z (%) = 285 (67) [M^+], 284 (100), 270 (35), 256 (22), 254 (21) 242 (17), 218 (22), 203 (61), 190 (36). MS (ESI): 286.2 $[M+H]^+$.

(*E*)-**19:** R_f 0.39 (Et_2O/MeOH 3:1); m.p. 109-112 °C; ^{1}H NMR (400 MHz): δ = 6.58 (s, 1H, 8-H), 6.56 (s, 1H, 11-H), 5.29 (q, *J* = 6.9 Hz, 1H, MeC*H*), 5.06 (dt, *J* = 2.3, 1.1 Hz, 1H, =CH*H*), 4.75 (br d, *J* = 2.3 Hz, 1H, =CH*H*), 4.41 (br s, 1H, 11b-H), 3.84 (s, 3H, OCH_3), 3.81 (s, 3H, OCH_3), 3.20 (dt, *J* = 12.1, 5.5 Hz, 1H, 6-H_a), 3.05–2.83 (m, 4H, 6-H_b + 4-H + 7-H_a), 2.69 (dt, *J* = 16.2, 5.6 Hz, 1H, 7-H_b), 2.46–2.37 (m, 1H, 3-H_a), 2.36–2.27 (m, 1H, 3-H_b), 1.76 (dd, *J* = 6.8, 07 Hz, 3H, =CCH_3) ppm. ^{13}C NMR (100 MHz): δ = 147.7 (s) and 146.8 (s) (C-9 and C-10), 143.1 (s, C-1), 138.9 (s, C-2), 126.4 (s, 2C, C-7a + C11a), 123.3 (d, =*C*H), 112.2 (t, =*C*H_2), 111.4 (d, 2C, C-8 + C-11), 66.2 (d, C-11b), 55.9 (q, O*C*H_3), 55.8 (q, O*C*H_3), 51.3 (t, C-4), 47.6 (t, C-6), 34.4 (t, C-3), 26.0 (t, C-7), 14.2 (q, =C*C*H_3) ppm. IR: ν = 3079, 3007, 2938, 2837, 1607, 1513, 1465, 1256, 1227 cm^{-1}. MS (70 eV, EI): *m*/z (%) = 285 (72) [M^+], 284 (100), 270 (44), 256 (11), 254 (9) 242 (7), 218 (10), 203 (64), 190 (45).

(Z)-**19:** ^{1}H NMR (400 MHz) detectable signals in the spectrum of the *E/Z* mixture: δ = 5.96 (q, *J* = 7.0 Hz, 1H, MeC*H*), 5.02 (br s, 1H, 11b-H), 4.89 (t, *J* = 2.1 Hz, 1H, =CH*H*), 4.65 (t, *J* = 2.1 Hz, 1H, =CH*H*) ppm.

Mixture of isomers: $C_{18}H_{31}NO_3$ (285.38): calcd. C 75.76, H 8.12, N 4.91; found C 75.37, H 8.42, N 4.70.

*(1S,2S,8aS)-1,2-Di-tert-butoxy-7-methyl-8-vinyl-1,2,3,5,6,8a-hexahydroindolizine (**20**)*

A mixture of VCP **15** (29.6 mg, 0.1 mmol) and $Rh(PPh_3)_3Cl$ (10 mg, 0.01 mmol) and TFE (0.09 mL; degassed with N_2 prior to use) in toluene (1.71 mL; distilled from Na/benzophenone and degassed with N_2 prior to use) in a sealed vial was irradiated in a microwave reactor at 130 °C for 3 h. Chromatography on silica gel (eluent: EtOAc/MeOH (NH_4OH 1%) 50:1) of the concentrated methanolic solution afforded **20** (15.7 mg, 53%).

20: R_f 0.31 [EtOAc/MeOH(NH_4OH 1%) 15:1]; $[\alpha]_D^{27}$= +33.6 (*c* = 1.147, CH_2Cl_2); ^{1}H NMR (200 MHz): δ = 6.58 (dd, *J* = 17.9, 11.4 Hz, 1H, =C*H*), 5.25 (dd, *J* = 17.9, 1.4 Hz, 1H, =C*H*H), 5.10 (dd, *J* = 11.4, 1.4 Hz, 1H, =C*H*H), 4.03–3.87 (m, 2H, 1-H + 2-H), 3.71 (br s, 1H, 8a-H), 3.25 (dd, *J* = 10.7; 5.9 Hz, 1H, 3-H_a), 3.07–3.71 (m, 3H, 3-H_b + 5-H), 2.38–2.19 (m, 1H, 6-H_a), 1.97–1.82 (m, 1H, 3-H_b), 1.78 (s, 3H, 7-CH_3), 1.20 (s, 9H, *t*-Bu), 1.15 (s, 9H, *t*-Bu) ppm. ^{13}C NMR (50 MHz): δ = 137.3, 132.3 (s, C7, C-8), 133.4 (d, =*C*H), 114.5 (t, =*C*H_2), 83.3 (d, C-1), 79.0 (d, C-2), 74.1 (s, *C*Me_3), 73.1 (s, *C*Me_3), 64.5 (d, C-8a), 58.2 (t, C-3), 45.8 (t, C-5), 29.0 (q, 3C, CH_3), 28.8 (q, 3C, CH_3), 27.9 (t, C-6), 19.8 (q, 7-CH_3) ppm. IR: ν = 3028, 2971, 1604, 1462, 1391, 1363, 1183 cm^{-1}; MS (70 eV, EI): *m*/z (%) = 307 (3) [M^+], 250 (46), 234 (2), 194 (20), 135 (100), 57 (37). HRMS: calcd for $C_{19}H_{34}NO_2$ $[M+H]^+$ 308.25841, found 308.25808.

*(3aR,9aS,9bS)-2,2,8-Trimethyl-9-vinyl-3a,4,6,7,9a,9b-hexahydro[1,3]dioxolo[4,5-a]indolizine (**21**)*

A mixture of VCP **17** (10.5 mg, 0.045 mmol), $Rh(PPh_3)_3Cl$ (4 mg, 0.05 mmol) and TFE (0.1 mL; degassed with N_2 prior to use) in toluene (1.9 mL; distilled from Na/benzophenone and degassed with N_2 prior to use) in a sealed vial was irradiated in a microwave reactor at 110 °C for 3 h and 30 min. Chromatography on silica gel [eluent: CH_2Cl_2/MeOH (NH_4OH 1%) from 30:1 to 10:1] of the concentrated crude mixture afforded **21** (3.6 mg, 34%).

21: R_f 0.28 [CH_2Cl_2/MeOH (NH_4OH 1%) 15:1]; $[\alpha]_D^{27}$= +81.1 (*c* = 0.504, CH_2Cl_2); ^{1}H NMR (200 MHz): δ = 6.71 (dd, *J* = 18.0, 11.4 Hz, 1H, =C*H*), 5.27 (d, *J* = 18.0 Hz, 1H, =C*H*H), 5.12 (d,

J = 11.4 Hz, 1H, =C*H*H), 4.68–4.60 (m, 1H, 3a-H), 4.55 (dd, *J* = 6.5, 1.9 Hz, 1H, 9b-H), 3.89 (br s, 1H, 9a-H), 3.08–2.80 (m, 4H, 4-H + 6-H), 2.50–2.26 (m, 1H, 7-H_a), 1.89–1.65 (m, 1H, 7-H_b), 1.79 (br s, 3H, 8-CH_3), 1.57 (s, 3H, OCH_3), 1.33 (s, 3H, OCH_3) ppm. ^{13}C NMR (50 MHz): δ = 133.0 (s) and 129.9 (s) (C-8 and C-9), 132.3 (d, =*C*H), 114.2 (s, 2-C), 113.7 (t, =CH_2), 84.7 (d, C-9b), 79.0 (d, C-3a), 65.7 (d, C-9a), 54.7 (t, C-4), 44.3 (t, C-6), 26.8 (q, 8-CH_3), 26.3 (t, C-7), 24.9 (q, OCH_3), 19.7 (q, OCH_3) ppm. IR: ν = 3042, 2936, 1636, 1381, 1208, 1051 cm^{-1}; MS (70 eV, EI): *m*/z (%) = 235 (20) [M^+], 234 (14), 220 (7), 176 (12), 135 (100), 120 (21), 106 (21), 91 (12), 79 (13). HRMS: calcd for $C_{14}H_{22}NO_2$ $[M+H]^+$ 236.16451, found 236.16446.

References

1. de Meijere, A.; Kozhushkov, S. I.; Späth, T. *Org. Synth.* **2002**,*78*, 142–151.
2. a) Brandi, A.; Garro, S.; Guarna, A.; Goti, A.; Cordero, F. M.; De Sarlo, F. *J. Org. Chem.* **1988,** *63*, 2430–2434; b) Yamazaki, S. *Bull. Chem. Soc. Jpn.* **1997**, *70*, 877–883; c) Zhao, B.-X.; Yu, Y.; Eguchi, S. *Org. Prep. Proced. Int.* **1997**, *29*, 185–194.
3. Zorn, C.; Anichini, B.; Goti, A.; Brandi, A.; Kozhushkov, S. I.; de Meijere, A.; Citti, L. *J. Org. Chem.* **1999**, *64*, 7846–7855.
